# Mechanism of water transport in graphene oxide laminates

**DOI:** 10.1039/c6sc03909j

**Published:** 2016-11-29

**Authors:** Junjiao Deng, Yi You, Heriberto Bustamante, Veena Sahajwalla, Rakesh K. Joshi

**Affiliations:** a Center for Sustainable Materials Research and Technology , School of Materials Science and Engineering , University of New South Wales , Sydney , NSW 2052 , Australia . Email: veena@unsw.edu.au ; Email: r.joshi@unsw.edu.au; b Sydney Water , Parramatta , NSW 2159 , Australia

## Abstract

It is understood that nano-channels of graphene oxide membranes have a water flow mechanism which is similar to the water flow inside carbon nanotube pores. The water transport mechanisms recently proposed by various researchers suggest that membranes composed of graphene oxide laminates could be regarded as an assembly of many tiny carbon nanotubes stacked together with attached functional groups as spacers.

## Abstract 2

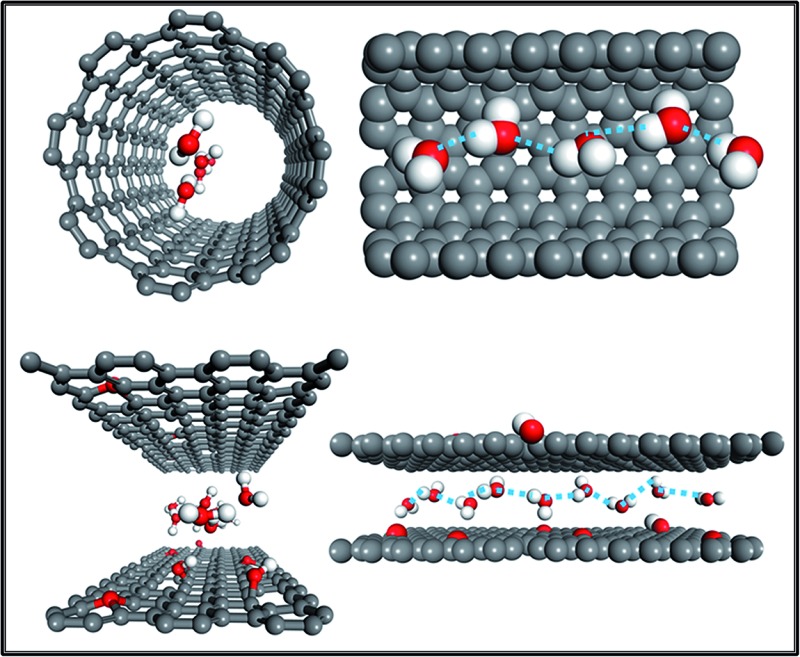
The water transport mechanisms recently proposed by various researchers suggest that membranes composed of graphene oxide laminates could be regarded as an assembly of many tiny carbon nanotubes stacked together with attached functional groups as spacers.

Graphene oxide (GO) with atomic thickness is a promising candidate for nanofiltration membranes because of its tunable chemical and physical properties, possibility to have high water flux and selective permeation.^1–4^ Present research reveals that in GO membranes, water molecules prefer to diffuse in non-oxidized hydrophobic nano-channels composed of stacked GO flakes.^1,2^ This fast water flow in the channels of GO membranes is suggested to be similar to that in carbon nanotube (CNT) membranes, in which water molecules move freely in the hydrophobic tubes.^2,3,5,6^ Additionally, GO laminates possess extra oxidized regions with varied functional groups and structural defects.^1,4^ However, it has been proven that the pristine hydrophobic region is the main contributor to high water flux.^1,2^ Herein, the question arises – can GO membranes be regarded as an assembly of numerous tiny broken CNTs stacked together with attached functional groups as spacers? This hypothesis dictates that the mechanism of water flow in CNT membranes explained previously can contribute towards understanding the mechanism of water transport in GO membranes as laminates. The purpose of this article is to relate the mechanism of water transport through GO laminates to that through the pores of pure CNTs.

We know that the GO membrane is an assembly of GO flakes stacked together into a network of nano-channels with *d*-spacing (0.83 nm). This can be further expanded under hydrated conditions, owing to the oxidized regions with oxygen-containing functional groups attached to the GO flakes.^3,7,8^ As Fig. 1 illustrates, there are three conditions constituting the water pathway in GO membranes: across defect pores^9–11^ (Fig. 1b), through inter-edge areas^9,10^ (Fig. 1c) and inside hydrophobic nano-channels, which is the most common^1,2^ (Fig. 1d). Compared with the other two conditions, the last one is more energy-favorable for water molecules to flow inside planar narrow channels provided by non-oxidized regions on the GO flakes.^1,8^

**Fig. 1:**
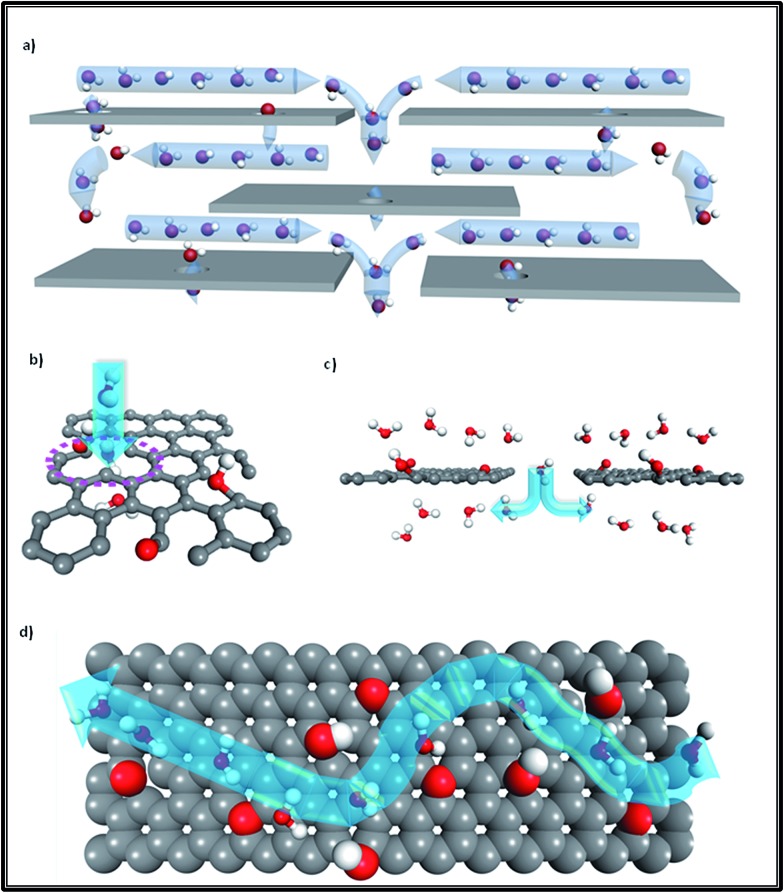
(a) Overview of the water transport mechanism for GO laminates; (b) transport through a defect pore; (c) transport *via* jumping from one channel to another channel through the inter-edge area; (d) top view of water molecules diffusing in a planar channel (the upper GO flake of this channel was cut out for better visualization). Grey color: carbon atoms; red color: oxygen atoms; white color: hydrogen atoms; blue arrows show the water flow direction. Adapted with permission from ref. 1 (B. Mi, *Science*, 2014, **343**, 740–742). Copyright 2014 The American Association for the Advancement of Science.

To study nano-fluidic behaviour, the slip length is employed to describe the boundary conditions when a specific pore radius is able to provide zero velocity at a supposed pore wall.^5^ So, figuring out the value of the slip length can help in detecting the degree of water molecules interacting with the nano-channels and clarifying the conditions under which fast water transport can be achieved.^12^ Theoretical studies have shown that water has a dramatically large slip length (up to 67 ± 45 nm) on a graphene surface,^13,14^ while the non-oxidized nano-channels are hydrophobic like a pristine graphene surface. Hence, friction-free water transport could be achieved in non-oxidized sections of GO nano-channels.^4,15^ Similarly to CNT membranes, when the slip length is much larger than the pore radius of the CNT, the wall of the CNT is nearly frictionless, thus resulting in a high water velocity.^5^ Especially for a long CNT, the flow rate is exclusively determined by the slip length.^5,16^

Additionally, as Fig. 2c shows, monolayer water molecules are observed in a cylinder plug-like formation when undergoing transport in GO non-oxidized nano-channels, just like that found in single CNTs (see Fig. 2a).^2,17^ It is known that water diffusion follows three motion mechanisms.^18^ The first is the Fickian-type, in which water molecules can collide with each other in the direction of movement with disordered molecular motion. The second is the single-file type in which a narrow space confines water molecules into a one chain configuration to avoid colliding with each other, and the last is a ballistic-type, in which the motion of confined water molecules is well-ordered.^18^ The second type of water motion can be found in the hydrophobic narrow channels of GO membranes, as Fig. 2d shows, in which single-file water molecules possess ordered H-bonds and reduced free energy.^17,19^ Similarly, when water molecules undergo transport in the narrow tubes of CNT membranes (*R*_threshold_ < 0.5 nm), the transition of water is changed from a continuum to a sub-continuum and the friction coefficient almost disappears due to the structure of water being transformed into a single-file configuration (see Fig. 2b) with long-lasting H-bonds.^20–23^ Moreover, a recent study observed that the confinement of water molecules in hydrophobic nano-capillaries can not only put them into a one-chain configuration but can also convert them into ice crystallites.^24^ Monolayer ice with high crystallinity is very mobile and has extremely weak interactions with a hydrophobic surface, which explains well the rapid water transport in the nano-channels of both GO membranes and CNT membranes.^24^

**Fig. 2:**
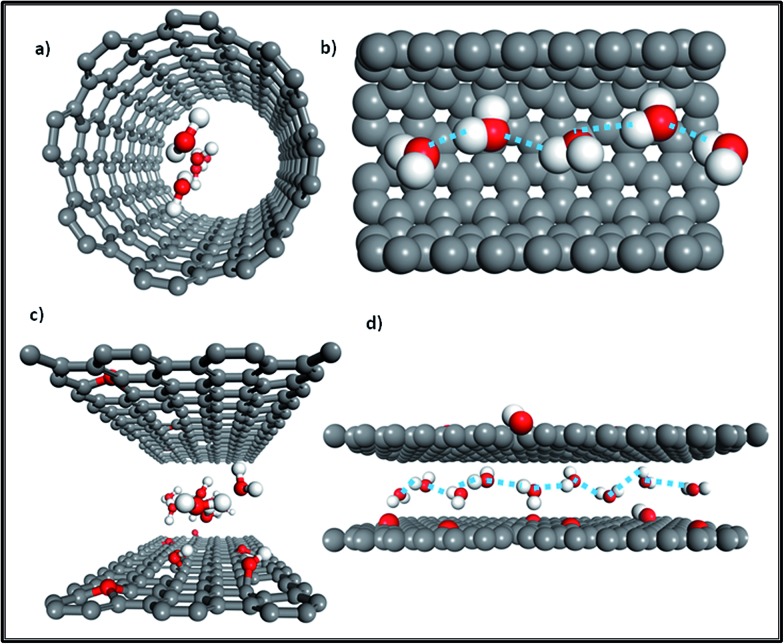
(a) Front view of the water molecule distribution in a CNT; (b) side view of water molecules confined in a single-filed configuration (a section of the tube was cut out for better visualization); (c) front view of water molecules diffusing in the nano-channels of GO membranes; (d) side view of water molecules in a single-file configuration moving in nano-channels (grey color: carbon atoms; red color: oxygen atoms; white color: hydrogen atoms; the dashed line represents ordered long-lasting H-bonds). Adapted with permission from ref. 23 (K. Falk, F. Sedlmeier, L. Joly, R. R. Netz and L. Bocquet, *Nano Lett.*, 2010, **10**, 4067–4073). Copyright 2010 American Chemical Society.

As discussed above, water molecules can freely move inside hydrophobic nano-channels, but they need to transversely flow through the inter-edge space from one hydrophobic channel to another as shown in Fig. 1c.^10,15^ Thus, water molecules must overcome an energy barrier when ‘jumping’ from one channel to the next. The significance of the energy barrier has been addressed well by Boukhvalov *et al.* ^10^ The authors suggested that the water molecules, while forming ice, will undergo a melting transition at the edges of the GO flakes as well as in the oxidized areas in the GO on achieving an ideal interlayer distance for the formation of bilayer ice.^10^ They further mentioned that compared with the first layer of ice, the second layer of ice is more energy-favorable for flowing along the plane to the edge of the GO flakes.^10^ Nevertheless, it has been discovered that the interaction between water molecules and other water molecules in the channel can effectively minimize the permeation barrier for water.^19,25^ Likewise, though water is allowed to freely flow inside the CNT tube, it encounters a significant energy barrier at both the entrance and exit of the tubes, including the change in free energy of each water molecule for breaking two H-bonds once they have entered the tube.^20,26^ Also, the ordered H-bonds between the water molecules inside the tubes allow the free energy to be much lower than that of the water outside the tubes, which consequently reduces the energy barrier for water molecules entering the tubes.^23^ Besides the transverse flow path, it has been seen that the oxidized region in the GO membrane is another limiting factor for water flow.^1,4,27^ The oxygenated hydrophilic groups at either the basal planes or edges of the GO flakes tend to interact strongly with water molecules, which may block them and reduce the water flux.^4,27^ Interestingly, some studies share different opinions, in which hydrophilic groups are also capable of promoting water permeation; specifically, the carboxyl groups at the edges of flakes and the hydroxyl groups at defect pores.^28,29^ Because H-bonds can be formed between the –OH part in those functional groups with water molecules, this results in ideal entropic conditions for water to either enter nano-channels or pass through defect pores.^28,30^ In addition, oxygen-containing groups such as hydroxyl, carboxyl and epoxy in amorphous regions possess a large content of distorted sp^3^ C–O bonds, resulting in nano-wrinkles and defect pores in GO flakes which offer initial passages through which water can diffuse into GO membranes.^9–11,30^ In general, the hydrophobic area is the most favorable pathway for water, whereas if the positions and quantity of hydrophilic groups were well-managed, oxidized regions could also contribute to the high water flux.^27,29^

The capillary force provided by nano-channels is identified as by far the main driving force to draw water inside GO membranes.^1–4^ Besides the theory of capillary force, a new aspect named ‘breathing’ was proposed as another factor prompting GO to suck water continuously.^31^ This discovery was made on the basis of the theory developed by Poynor *et al.* that says a low-density depletion layer can be thermodynamically formed once water passes through a hydrophobic surface.^26,32^ Because the density of water in hydrophobic nano-channels is much lower than that in bulk water, this consequently leads to the pressure inside the nano-channels being smaller than that of bulk water.^31^ Therefore, this pressure difference can ‘push’ water into GO membranes. Neek-Amal *et al.* ^33^ found that the shear viscosity of nano-confined water is reduced on decreasing the density of nano-confined water.^33^

In the case of CNT membranes, the depletion layer on the surface of the tube is in the range of monolayer water thickness with a concentration of water molecules (2.8 M) considerably lower than that of the bulk water concentration (55 M).^26^ Moreover, the water structure, including the H-bonding and OH-bonds’ orientation, in the depletion layer directly influences the water flow rate in CNTs.^16,20,26^ The free OH bonds orientated towards the CNT wall can make the H-bonds between water molecules in the depletion layer weak enough to allow super-fast water flow to be achieved.^16,26^ Nevertheless, to achieve wider scope in GO membrane applications, more attention should be paid to how to improve the water flux towards a mass level, which could be achieved by tailoring the pore size. One possible way is to modify the GO synthesis method to introduce functional groups such as a stable surfactant that can be favorable for both the water flux and selectivity of the membranes.

In summary, based on comparison of the water flow mechanisms in CNT membranes and GO membranes, the pristine ‘empty’ nano-channels in GO membranes can be regarded as an assembly of broken tiny CNTs. Their identical characteristic is having hydrophobic narrow spaces that confine water molecules into a single-file configuration with high crystallinity to achieve an ultra-fast flow velocity. In addition, both of them have a depleted water layer present on the hydrophobic surface, which further clarifies why GO membranes can continuously suck water in, besides the theory of capillary force. Additionally, the oxidized spacers in GO based membranes play a crucial role. These oxidized spacers could enhance water permeation if they were in a suitable amount and position. Further research is needed to fully explore the water flow mechanism in GO membranes for practical applications in desalination and water purification purposes.
